# Theranostic barcoded nanoparticles for personalized cancer medicine

**DOI:** 10.1038/ncomms13325

**Published:** 2016-11-10

**Authors:** Zvi Yaari, Dana da Silva, Assaf Zinger, Evgeniya Goldman, Ashima Kajal, Rafi Tshuva, Efrat Barak, Nitsan Dahan, Dov Hershkovitz, Mor Goldfeder, Janna Shainsky Roitman, Avi Schroeder

**Affiliations:** 1 Department of Chemical Engineering, Laboratory for Targeted Drug Delivery and Personalized Medicine Technologies, Technion—Israel Institute of Technology, Haifa 3200003, Israel; 2 Infrastructure Unit, Life Science and Engineering Center, Technion—Israel Institute of Technology, Haifa 3200003, Israel; 3 Clinical Pathology Unit, Rambam Medical Center, Haifa 3525408, Israel

## Abstract

Personalized medicine promises to revolutionize cancer therapy by matching the most effective treatment to the individual patient. Using a nanoparticle-based system, we predict the therapeutic potency of anticancer medicines in a personalized manner. We carry out the diagnostic stage through a multidrug screen performed inside the tumour, extracting drug activity information with single cell sensitivity. By using 100 nm liposomes, loaded with various cancer drugs and corresponding synthetic DNA barcodes, we find a correlation between the cell viability and the drug it was exposed to, according to the matching barcodes. Based on this screen, we devise a treatment protocol for mice bearing triple-negative breast-cancer tumours, and its results confirm the diagnostic prediction. We show that the use of nanotechnology in cancer care is effective for generating personalized treatment protocols.

## Abstract (web summary)

Determining the most effective treatment for each cancer patient is a key challenge in cancer therapy. In this article, the authors show, in a mouse model of breast cancer, that DNA barcoded nanoparticles can be used for pre-screening the efficacy of anticancer drugs.

## Graphical abstract

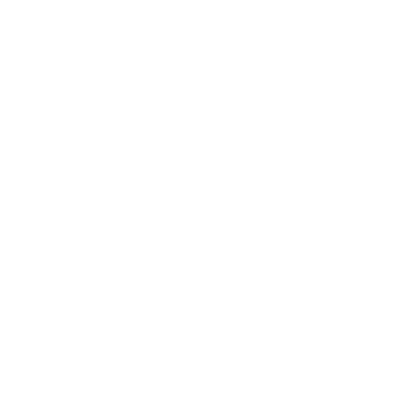

In cancer, a key challenge is determining the most effective medicinal treatment for each patient. In the past, after a short chemotherapeutic cycle, biopsies were taken from the patient's tumour and examined histologically for tumour response^1^. Imaging technologies later enabled the tracking of treatment efficacy, based on tumour regression/progression, vasculature proliferation and metabolic activity^2^,^3^. However, even advanced imaging modalities have their restrictions, including overlapping spectral bandwidths of the contrast agents and poor light penetration in tissues^4^. This limits the assessment of the therapeutic activity at a single-cell level, or at differentiating between the therapeutic activities of multiple drugs inside the tumour. In recent years, genetic signatures and patient-specific biomarkers have advanced the field of personalized medicine^5^. Yet gaps in our understanding of the complex genetic and epigenetic pathways^6^ leave room for complementary technologies that probe for drug activity inside the tumour cells.

To prescreen multiple drugs for their anticancer activity, *in vitro* and *in vivo* assays were developed^7^,^8^. These tests are based on tumour tissue that is biopsied from a patient and then either grown in a dish (*in vitro*) or implanted into immune-deficient mice (*in vivo*)^7^. Subsequently, the cells, or the mice, are treated with various drugs to predict the most efficient treatment. Although these methods are available, controversy regarding the accuracy of the *in vitro* approach^9^, as well as the lengthy *in vivo* implantation process, have hampered wide clinical implementation^10^.

Nanomaterials are becoming important medical tools, granting therapeutic precision and diagnostic functionality that cannot be attained using methods of larger scale^11^,^12^. More than 40 nanomedicines have already been approved for clinical use and experimental nanotechnologies have the potential to revolutionize diagnosis and care^13^,^14^.

Therapeutic nanoparticles are effective carriers of a wide range of medicines, including small molecules, nucleic acids and proteins^15^. These nanoparticles can target diseased tissues, including tumours and metastasis^16^, as well as track the biodistribution of drugs at real time^17^,^18^. More specifically, liposomes, vesicles with an inner aqueous core surrounded by a lipid bilayer, are clinically approved drug carriers^11^. When injected intravenously, 100 nm liposomes accumulate preferentially in solid tumours, penetrating through defects in the endothelial wall of tumour capillaries^19^. This phenomenon is known as passive targeting, or as the enhanced permeability and retention effect^20^. Although not all tumours display permeable vasculature, the enhanced permeability and retention effect has been demonstrated pre-clinically and clinically in metastatic breast^21^,^22^ and ovarian cancers^23^,^24^, melanoma^25^,^26^, AIDS-related Kaposi sarcoma^27^,^28^ and other diseases^29^,^30^,^31^.

For diagnostic applications, synthetic DNA has been suggested as a useful probe^32^,^33^,^34^,^35^,^36^. A DNA barcode is a sequence of nucleotides that can be decoded using one or more technologies, such as PCR and sequencing. These assays have several benefits, including an extremely sensitive detection threshold (∼30 attomolar), high versatility and a short analysis time compared with other assays^36^,^37^,^38^,^39^.

Herein, we sought to harness these advantages of nanotechnology, combined with DNA barcodes, to screen anticancer drugs for their therapeutic potency inside the patient's tumour (Fig. 1). Screening active agents inside a patient's body is adapted conceptually from allergy tests, in which minuscule doses of allergens are injected subdermally. Unlike allergy tests, which elicit a visible reaction on the skin, detecting the activity of multiple medicines inside the tumour cells adds another level of complexity to the process. Therefore, we load nanoparticles with anticancer medicines and corresponding DNA barcodes, enabling us to track the drugs that entered each tumour cell and follow their potency more precisely. Using the proposed method, we were able to advise an efficient, personalized therapeutic protocol within less than 72 h.

## Results

### Barcoded nanoparticles

The nanoscale probes, used for gauging drug activity, were constructed of 100 nm liposomes (Supplementary Fig. 1), loaded with minuscule amounts of anticancer agents and corresponding synthetic DNA strands. DNA offers unlimited barcoding possibilities, with extremely sensitive detection modalities^40^. We chose to work with 50 to 120 base-pair (bp)-long double-stranded DNA barcodes. At these lengths, DNA is less susceptible to endogenous degradation by DNase^41^ or to eliciting immune responses^34^.

**Figure 2:**
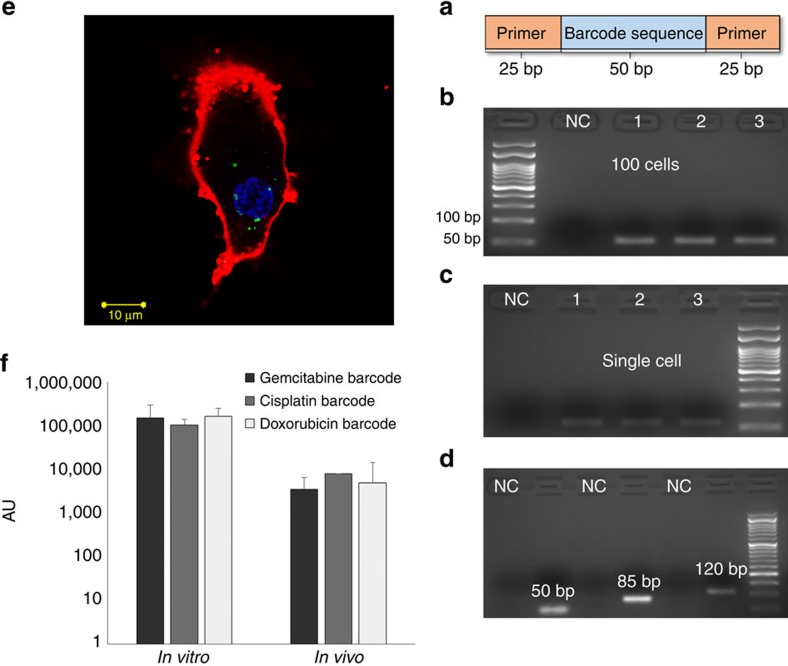
DNA was used as a barcode for labelling and detecting nanoparticles in single cells. Synthetic DNA strands were embedded in 100 nm liposome together with a corresponding drug. (**a**) The barcodes were constructed to be in the range of 50 to 120 bp long, and detected using PCR and sequencing. Barcoded nanoparticles were taken up spontaneously by triple-negative breast cancer cells in culture and in tumours. (**b**) Gel electrophoresis of PCR-amplified barcodes derived from 100 cells, and (**c**) from a single cell. (**d**) Different strand lengths of barcodes (50, 85, 120 bp) can be detected within a single-cell suspension. Negative control wells are designated NC, and repeats are numbered 1–3. (**e**) A confocal microscopy image of uptake of BNPs by a triple-negative breast cancer 4T1 cell labelled for membrane (red), nucleus (blue) and DNA barcodes (green). The single-cell uptake of barcoded nanoparticles is not affected by the cargo (**f**). Barcoded nanoparticles, all 100 nm in diameter but loaded with different cargo, were added to triple-negative breast cancer cells in culture, or injected intravenously into tumour-bearing mice. To ensure the uptake of multiple particles per cell, a dose 1,000 times higher than that used for the diagnostic procedure was administered. The cells were collected from the dish (after 24 h), or a biopsy was taken from the tumour (after 48 h). The tissue was then dissociated, and 60 individual cells were examined for the presence of each of the different barcodes. Each cell contained a similar number of each of the barcodes, indicating that the internal payload of the nanoparticles does not affect their cellular uptake. The data were calculated as the mean±s.e.m. of *n*=60.

The barcode has three regions, which allowed us to detect and quantify it at the single-cell level; a forward primer (20 bp), a code region (10–80 bp) and a reverse primer (20 bp). Varying the primers, or altering the barcode sequence and length, enables detecting the barcode by sequencing, PCR and gel electrophoresis (see the ‘Methods' section, Fig. 2a,d). In addition, the sequences were kept nonspecific, biologically noncoding and were designed not to form major secondary structures (for a full sequence list, see Table 1 in the ‘Methods' section). Each nanoparticle was loaded with approximately 15 barcodes.

### Barcoded nanoparticles detected in a single cancer cell

When the barcoded nanoparticles were added to triple-negative 4T1 breast cancer cells in culture, they were taken in readily by the cells. To detect the barcodes, we washed the cells thoroughly and sorted them, by FACS, to clusters of 1,000, 100, 10 or single cell. The cells were lysed, and the barcodes were extracted and amplified using real-time (RT) –PCR. Both the carrier (liposome) and the barcodes were detected at a single-cell level, using both microscopy and biochemical assays (Fig. 2b,c,e). After the extraction process, the barcodes were also sequenced, to validate the specificity of the process. Free (non-particulate) DNA barcodes were not taken in by the cells; indicating that the barcodes found intracellularly were carried into the cell by the nanoparticle (Supplementary Fig. 2).

Barcoded nanoparticles were loaded with an anticancer drug or with a control compound (see the ‘Methods' section). The number of barcodes found intracellularly corresponded to the concentration of drug found inside the cells. Specifically, HPLC analysis of the drug found in cells, correlated with the RT–PCR quantification of the cellular barcodes (Supplementary Fig. 3a).

### Barcoded nanoparticles accumulate in the tumour

**Figure 3:**
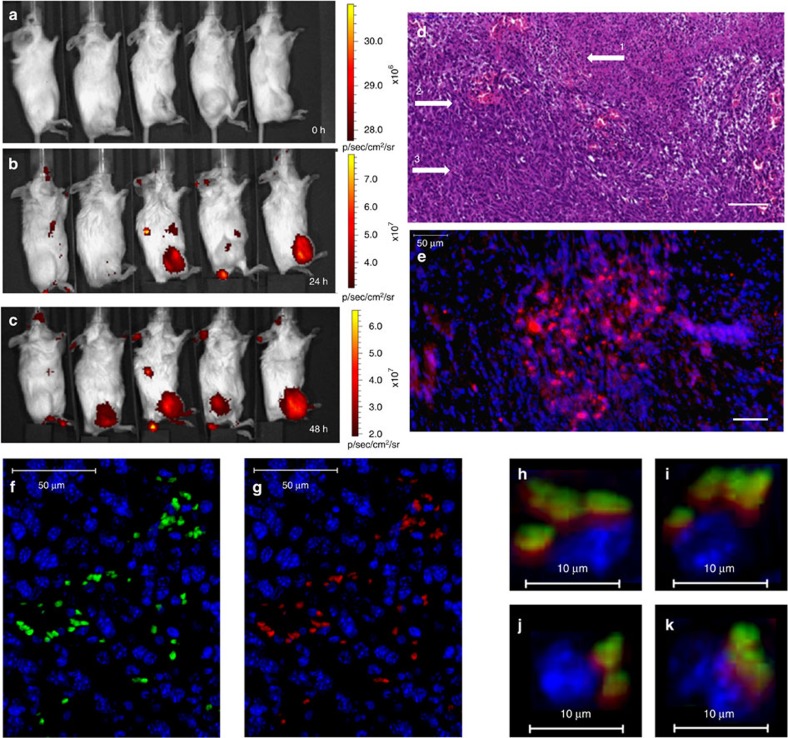
Barcoded nanoparticles accumulate in triple-negative breast cancer tumour cells. Barcoded nanoparticles, containing the diagnostic imaging agent indocyanine green (ICG), were injected intravenously to BALB/c mice bearing a tumour in their hind leg. The animals were imaged over 48 h: before the injection (**a**), 24 h (**b**) and 48 h after the injection (**c**). The fluorescent ICG intensity is presented by the red–yellow scale bar on the right side of each sub-figure. The left mouse on each figure is a healthy control and all the animals to the right are tumour-bearing mice; all animals were injected with 150 μl of barcoded ICG nanoparticles. After 48 h, the tumour was resected and examined histologically. The tumour tissue was stained with haematoxylin and eosin stain (H&E) (**d**). The tumour is closely packed where three major parts can be observed: necrotic tissue (1), multiple blood vessels that contribute to the EPR effect (2) and cancerous cells (3). To detect the liposomal accumulation in the tumour tissue, an overlay fluorescent image is presented (**e**); the cell nuclei were stained with DAPI (blue) and the particles with rhodamine (red). To detect the barcoded nanoparticles in single cells within the tumour, the barcode was labelled with fluorescein (**f**, green) and the particle membrane was labelled with rhodamine (**g**, red). Co-localization of the barcodes and the particles can be seen inside single cells (**h**–**k**). The scale bar in the histology images represents 100 μm, in the fluorescent images 50 μm (**e**–**g**) and in the fluorescent single cells 10 μm (**h**–**k**).

We studied the biodistribution of nanoparticles in BALB/c mice bearing triple-negative breast cancer (TNBC, 4T1) xenografts. Barcoded liposomes, loaded with a diagnostic agent (indocyanine green, ICG), were injected intravenously, and the mice were imaged over a period of 48 h. We compared the preferential accumulation of the liposomes in the tumour site with the free dye (Fig. 3a–c). The free dye was observed in the tumour, spleen and liver, during the first 24 h, while the liposomal accumulation in the tumour increased over a period of 48 h (Supplementary Fig. 4). Histological analysis of the tumour showed that the nanoparticles were taken up by the tumour cells (Fig. 3d–k), which was also confirmed after extracting the barcodes from the tumour cells. To achieve this, the tumours were resected and dissociated enzymatically into single-cell suspension. We were able to detect 0.1% of the injected dose of the barcoded nanoparticles inside the biopsied tumour cells.

### Barcoded nanoparticles as a diagnostic tool

We used the barcoded nanoparticles to investigate the tumour sensitivity to drugs. All the barcoded nanoparticles had an identical, 100 nm structure, and varied only in their internal content. Three of the barcoded nanoparticles contained clinically approved chemotherapeutics: doxorubicin, cisplatin and gemcitabine. One contained caffeine, as a control compound, and one was a placebo (nanoparticles with a barcode and phosphate-buffered saline, PBS). To ensure the screening had no systemic effect, the total dose of all the injected barcoded nanoparticles combined was below 1/1,000th the therapeutic dose.

A cocktail containing a mixture of all five barcoded nanoparticles was injected intravenously to mice bearing 4T1 xenografts. A core biopsy was taken from the tumour 48 h later. This time frame ensured that the apoptotic cells remain intact for future analysis^42^. The biopsied tumour tissue was dissociated enzymatically into a single-cell suspension, and then sorted by FACS according to cell viability (live/dead, using a propidium iodide stain). The live and dead cells were collected separately, washed, lysed and the DNA barcodes were extracted. Then, each group's barcodes were amplified using RT–PCR. Barcodes found in the dead cells were characterized as active drugs, whereas barcodes found in the live cells were of nonactive drugs.

During the procedure, the animals did not lose weight or change their behavioural patterns; this can be explained by the low total dose of the administered drug.

**Figure 4:**
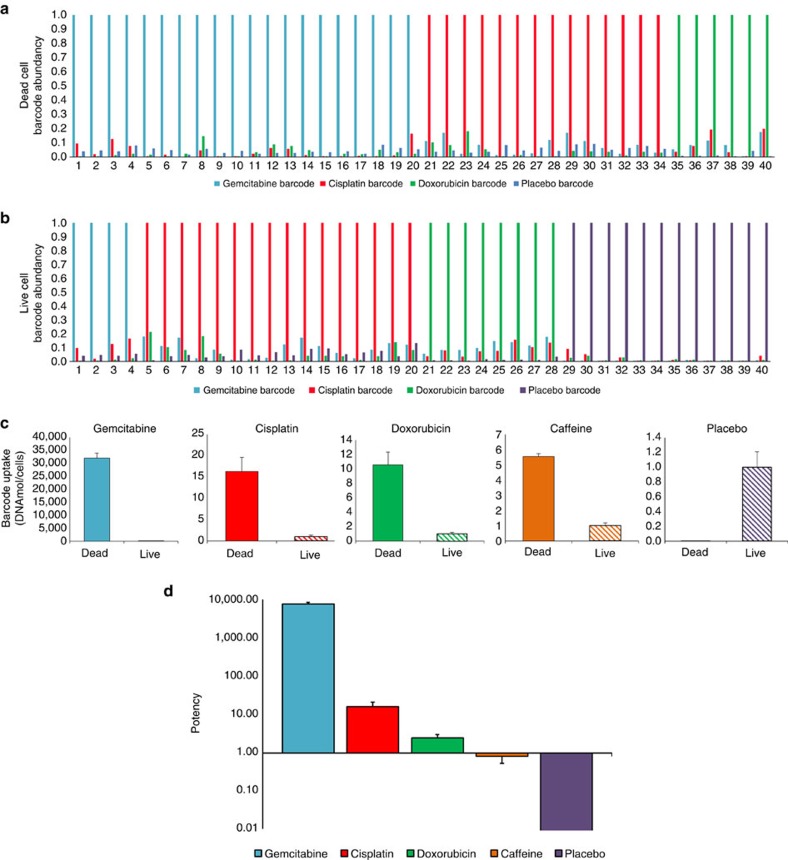
Predicting the therapeutic potency of multiple drugs using barcoded nanoparticles. Mice bearing triple-negative breast cancer tumours were administered five different barcoded nanoparticles, simultaneously. The nanoparticles contained either one of the anticancer agents: doxorubicin, gemcitabine or cisplatin, or were loaded with caffeine, or empty (barcode alone). The barcoded nanoparticle cocktail was injected intravenously and accumulated in the tumour cells over a period of 48 h. After 48 h, the tumour tissue was biopsied and dissociated into single cells. The cells were sorted according to their viability (live/dead) and the barcodes in each of these populations were extracted, analysed and quantified using RT–PCR and sequencing. The activity of each of the agents was measured at the single-cell level; data from 80 representative cells are shown (**a**,**b**). A noise level below 20% was set as the threshold for determining the activity of a single agent at the single-cell level. In addition, (**c**) the overall activity of each of the agents in the tumour was determined by analysing the barcode abundance in groups of at least three million live or dead cells. (**d**) To compare between the potency of the different drugs, a potency scale was plotted. The comparative potency is based on the ratio of barcodes found in the dead cells to those found in live cells. On the basis of this comparative diagnostic scale, a treatment protocol was devised. To ensure statistical significance, each screen was based on at least three million cells extracted from the tumour. The data were calculated as the mean±s.e.m. of *n*=6, in two independent experimental replicates.

We found a correlation between the drug activity and the presence of a barcode in the live or dead cells of the tumour (Fig. 4). As expected, both doxorubicin and cisplatin showed a positive therapeutic effect, while caffeine showed a minor therapeutic effect^43^,^44^. However, the most astonishing results came from gemcitabine and the placebo. Gemcitabine was found to be the most efficient drug based on its barcode distribution. The gemcitabine barcode accumulated 30,000 times more abundantly in the dead cells compared with live cells (Fig. 4c). In contrast, the placebo barcodes accumulated 3,000 times more abundantly in live cells compared with the dead cells.

To rank the drugs according to their therapeutic activity, we defined the potency parameter as: Barcode_Dead cells_/Barcode_Live cells_; the greater the ratio, the more potent the drug is in the patient. In accordance, gemcitabine was found to be the most potent drug and the placebo showed no therapeutic activity, Fig. 4d.

To ensure statistical significance and to reduce false positive hits of the drug activity analysis, we sought to have as many tumour cells as possible that contained only one type of barcode. Therefore, we limited the total number of injected barcoded nanoparticles. Our biodistribution analysis showed that 0.1% (which are 1/1,000) of the injected nanoparticles are found in the biopsied tumour cells. Furthermore, we were able to extract approximately 40,000 intact cells per mm^3^ tumour tissue. Therefore, the maximal number of the injected barcoded nanoparticles can be determined based on the tumour volume, and should not exceed × 40,000 (tumour volume, mm^3^) × 1,000. For example, for a 500 mm^3^ tumour, 2 × 10^10^ barcoded nanoparticles were injected, which correlate to a total liposomal dose of 0.005 mg drug per kg body weight.

### Treatment efficacy mirrors the diagnostic procedure

**Figure 5:**
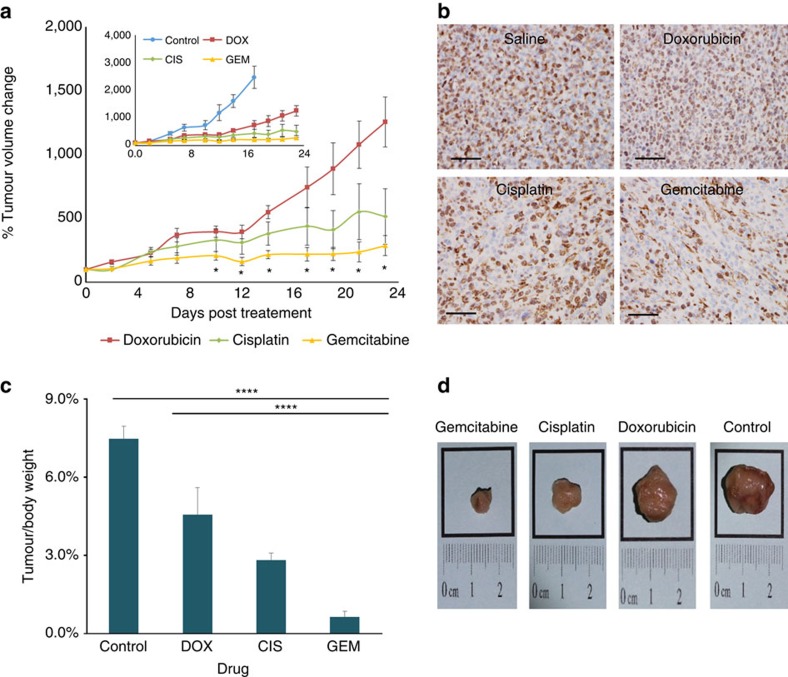
Treating mice according to the barcode analysis. Based on the barcoded nanoparticle drug screen, a treatment protocol was devised. Mice bearing triple-negative breast cancer tumours were administered doxorubicin, gemcitabine, cisplatin or saline (control). Tumour growth (**a**) was recorded and postmortem resection (**c**) and histology (**b**) of each of the groups was performed. Each group received a therapeutic weekly dose of chemotherapy—doxorubicin (5 mg kg^−1^), cisplatin (6 mg kg^−1^) or gemcitabine (125 mg kg^−1^), while the control group was administered saline. The tumours were resected 23 days after starting the treatment. Tissue slides (**b**) were immunohistochemically stained with rabbit monoclonal anti-Ki67 antibody to compare the proliferation rate of each group. These show a reduction in the proliferation rate in the gemcitabine-treated tumours compared with the other groups. The data were calculated as the mean±s.e.m. of *n*=6 per group; *P<0.01; ****P<0.0001. Differences between the two means were tested using an unpaired, two-sided Student's *t*-test. The efficacy of the treatment is also shown in the tumour size (**d**), thereby indicating the potency of the treatment. Before introducing the treatment, all the mice had the same average tumour size.

On the basis of the diagnostic analysis, we devised a treatment protocol. We administered gemcitabine, cisplatin, doxorubicin or saline to four groups of mice bearing TNBC tumours (six mice per group). The therapeutic outcome mirrored the prediction (Fig. 5). The tumour size of gemcitabine-treated mice was smallest and gemcitabine attenuated the growth of the tumours relative to doxorubicin, cisplatin and the control treatments (Fig. 5a,c,d). All the groups responded in a similar manner to the potency parameter; this was also exemplified by the Kaplan–Meier survival curve (Supplementary Fig. 5). Twenty-four days after beginning the treatment, the tumours were resected for tissue analysis. Interestingly, the histological examination demonstrated that tumours that showed clinical response to therapy were also less cellular and had lower mitotic counts. An immunohistochemical analysis (using anti-Ki67 antibody) demonstrated a lower proliferation index in tumours that received gemcitabine (40%) in comparison with those treated with doxorubicin (65%), cisplatin (85%) or saline (90%; Fig. 5b). In summary, the histological evaluation of gemcitabine-treated mice had a reduced proliferative state in comparison with the other groups, indicating improved prognoses also at the tissue level (Fig. 5b).

## Discussion

Personalized medicine aims to tailor treatments to accommodate each patient's unique disease presentation^45^,^46^. Since patients respond differently to medication; choosing the best drug for the individual patient, at the right time, is crucial for the success of the treatment. Common assays of personalized medicine involve genetic signatures and patient-specific biomarkers. However, gaps in our understanding of genetics and epigenetic pathways leave room for complementary diagnostic technologies that predict drug activity inside the tumour.

Our approach is to probe the patient's tumour for its sensitivity to anticancer medications, before beginning a treatment cycle. For this, we developed DNA-barcoded nanoparticles loaded with minuscule doses of various therapeutic agents. The nanoparticles were shown to target the tumour cells after being injected intravenously (Fig. 3), and the barcodes were used as mediators to correlate which drug is most therapeutically efficient. Double-stranded DNA offers unlimited barcoding possibilities, a relatively long half-life *in vivo* (Fig. 4)^41^, and quantitative detection modalities that far exceed analytical methods used for detecting a drug in tissue^40^. In addition, DNA cannot penetrate a cell without a vehicle, thereby ensuring that the cellular presence of a barcode was mediated by the nanoparticle. The barcode enabled us to assess the therapeutic sensitivity at various levels of detection, reaching even a single cell (Fig. 2b,c,e). Our analysis showed that approximately 0.1% of the intravenously injected nanoparticles are taken up by the tumour cells.

Theoretically, hundreds of different drugs/anticancer agents can be screened with the technology. The noise of this screen can rise due to uptake of several particles by an individual cell. To optimize the signal-to-noise ratio of the technology, there is a need to both minimize the number of particles per cell and to maximize the number of analysed cells. In fact, we found that injecting less than 4 × 10^7^ barcoded nanoparticles per cubic millimetre tumour, results in a single nanoparticle per cell. This takes into account the biodistribution of the nanoparticles and our ability to source approximately 40,000 intact cells from each cubic millimetre of dissociated tumour. To improve the predictive confidence, and to increase tumour-cell heterogeneity, we analysed three million cells from each tumour. Furthermore, after sorting the cells according to their viability and barcode type, we insisted on having at least 100,000 cells per efficacy subgroup (such as dead cells with gemcitabine or live cells with caffeine).

The disease model we chose to evaluate the technology in was TNBC. Owing to an absence of hormone receptors such as oestrogen and progesterone, nor overexpressed HER2, triple-negative tumours lack a clear medicinal treatment modality. TNBC also has poor prognosis, low survival and high recurrence rates. TNBC treatments usually involve systemic chemotherapy infusions^47^.

We used the barcoded nanoparticles to screen three clinically approved chemotherapeutics: doxorubicin, cisplatin and gemcitabine, all prescribed in advanced breast cancer^48^,^49^,^50^,^51^,^52^. Among the three, gemcitabine was found to be the most efficient drug, based on its barcode distribution in dead tumour cells. After gemcitabine, both doxorubicin and cisplatin showed a positive therapeutic effect as expected. The placebo barcode showed no therapeutic activity (Fig. 4d). As a diagnostic tool, the barcode distribution in cells correlated well with the therapeutic potency (live/dead, Fig. 4c), reducing false-positives. The rationale behind primarily finding barcodes of potent drugs in dead cells is that once a cell begins apoptotic processes it will not take up additional nanoparticles. Live cells, which took up ineffective drugs or placebo particles, were found alive in the tumour.

Then we tested the accuracy of the diagnostic prediction in a treatment mode. Tumour-bearing animals were treated with each of the drugs and the tumour progression was tracked. While nanoparticulate drugs enabled diagnostic prescreening at the single-cell level, the treatment was performed using an intravenous infusion of the free drugs (simulating the common clinical route of administration). Mice bearing breast cancer tumours were administered gemcitabine, cisplatin, doxorubicin or saline. The parameters of final tumour size, progression rate and proliferative state mirrored the intratumoral cellular prediction (Fig. 5).

To conclude, this study describes the development of a diagnostic nanotechnology that prescreens multiple drugs inside the tumour, with emphasis on cellular sensitivity. From a clinical perspective, our aim is to provide physicians with a new tool that matches each patient with the most potent medicine, during the different stages of disease to improve cancer care.

## Methods

### Liposome fabrication

Liposomes were composed of 55 mole% hydrogenated soybean phosphatidylcholine (HSPC; Avanti Polar Lipids, Alabaster, AL, USA), Mw 762 g mol^−1^; 5 mole% polyethylene glycol distearoyl-phosphoethanolamine (m2000PEG DSPE; Avanti), Mw 2,805 g mol^−1^; and 40 mole% cholesterol (Sigma-Aldrich, Revohot, Israel), Mw 386.65 g mol^−1^. Loading concentration of the lipid was 50 mM in a 5% w/v aqueous dextrose solution (5% DEX). To form liposomes, lipids were dissolved in pure ethanol, warmed to 65 °C and added to 1 ml of the 5% DEX. The liposomes were downsized by stepwise extrusion, using 400, 200, 100 and 50 nm pore-size polycarbonate membranes (GE Water and Process Technology, Osmonics) in a Lipex Extruder (Northern Lipids, Canada). After extrusion, the size was measured, using dynamic light scattering (ZetaSizer ZSP, Malvern Instruments, UK). The polydispersity index was between 0.04 and 0.08 and particle size 110±10 nm. In addition, the nanoparticles were characterized qualitatively using CryoTEM (‘Supplementary Methods').

### Drug-loaded barcoded nanoparticles

Desalted dsDNA oligos (Integrated DNA Technologies, IDT, Leuven, Belgium) ranging 50–120 bp (Table 1) were annealed at 60 °C and diluted to a concentration of 100 μM. The barcodes were inserted into the liposomes during the lipid hydration process by adding them to the aqueous phase at a DNA/lipid ratio of 1:20, respectively. At that point the liposomes were extruded to reach the final size. Non-encapsulated DNA was removed by dialysis, using a 10^6^ MW cutoff membrane (Spectrum Laboratories, Inc., USA).

Drug-loaded barcoded liposomes were prepared either by active (doxorubicin and gemcitabine) or passive (cisplatin, caffeine) loading. The barcodes were loaded as described above. Doxorubicin and gemcitabine were loaded into barcoded nanoparticles using an ammonium sulfate gradient, reaching a final concentration of 1 mg ml^−1^ according to Haran *et al*.^53^. Cisplatin was loaded in an NaCl environment according to Peleg-Shulman *et al*.^54^. Caffeine was dissolved using DMSO/ethanol (1/1, v/v) together with the lipids and then hydrated using 5% dextrose. The non-encapsulated drugs were removed by dialysis. When injected intravenously, the dose of all the drugs combined is held below 0.005 mg drug per kg body weight.

### Cellular uptake

*In vitro* experiments were conducted on 4T1 (triple-negative breast cancer, ATCC) murine cell lines and MDA-MB-231 (human breast cancer, ATCC) in culture (Roswell Park Memorial Institute medium; Sigma-Aldrich, Rehovot, Israel). Twenty-four hours after seeding the cells, 10% of the volume was replaced with the barcoded liposome solution and incubated for 24 h at 37 °C.

To test the barcode uptake, the cells were washed thoroughly with PBS, detached with trypsin, centrifuged at 400*g* for 5 min, and the DNA was extracted using a modified Bligh and Dyer assay^55^. Specifically, a chloroform:methanol:sample, ratio of 1:1:1 (v/v) mixture was prepared. The sample was centrifuged at 400*g* for 5 min, achieving phase separation, where the upper aqueous phase contained both the genetic and the synthetic dsDNA barcodes.

### PCR and RT–PCR

dsDNA barcodes were amplified by PCR in a thermocycler (LabCycler SensoQuest PCR, SensoQuest, Germany) and quantified by RT–PCR thermocycler (BioRad CFX96, Bio-Rad Laboratories Ltd., Israel). Cycling times: 5 min at 95 °C, (15 s at 95 °C, 45 s at 63 °C) × 40 cycles. Then, the solution was loaded on a 3% (w/w) agarose gel and separated for 25 min at 100 V (Wide Mini-Sub Cell GT Cell and PowerPac Basic Power Supply, Bio-Rad Laboratories Ltd., Israel). To estimate DNA band size, a DNA ladder was used (50 bp DNA ladder RTU, GeneDireX, Hy Laboratories, Israel). To quantify the barcodes, real-time PCR was used. Following DNA extraction, the strands were amplified and analysed using TaqMan Probe (qPCRBIO Probe Mix Lo-Rox+PrimeTime Mini qPCR Assay; IDT). Each barcode was independently amplified using specific probe and primers. Cycling times: 5 min at 95 °C, (15 s at 95 °C, 45 s at 63 °C) × 40 cycles. After PCR and DNA purification using a QIAquick PCR Purification Kit (Qiagen, CA, USA), the samples were sequenced (HyLabs, Jerusalem, Israel).

### Cell sorting

The cells were sorted using a flow-activated cell sorter (FACS; FACSARIA III, BD Biosciences, San Jose, CA, USA), and separated according to: (i) cell count—1,000, 100, 10 cells and a single cell; and (ii) viability condition—dead cells were labelled using propidium iodide (PI) assay^56^. Single cells were selected after defining a gate in FSC/SSC dot plot. To sort live/dead cells, a subpopulation in the single cells population was chosen. The dead cell's gate was defined for PI positive staining.

### Barcode encapsulation and uptake

The cells were seeded in optical plates (60 μ-Dish 3.5 cm, high‘−'ibidi‘', Madison, WI, USA) for 24 h, and then exposed for an additional 24 h to liposomes encapsulating fluorescently labelled dsDNA barcodes (labelled with 6-flourescin amitide—IDT). Then, the cells were washed thoroughly and the cell membrane was stained with DID (1,1′-dioctadecyl-3,3,3′,3′-tetramethylindodicarbocyanine, 4-chlorobenzenesulfonate salt, Biotium, USA) or Hoechst (Life Technologies, USA) for nuclei labelling. The cells were imaged using confocal microscopy (LSM-700, Zeiss, Germany) equipped with Axiovision software, and +cell observer (Zeiss). The images were analysed using Imaris software (Bitplane, Zurich, Switzerland).

### Tumours

All the animal studies were approved by, and complied with, the institutional and ethical committee at the Technion. Animal well-being was monitored daily by the researchers and staff veterinarians.

A total 10^6^ 4T1 cells, suspended in 100 μl PBS, were injected subcutaneously to 10-week-old BALB/c female mice (Harlan Laboratories Inc., Jerusalem, Israel). About 14 days later (allowing the primary tumour to reach 5 mm in diameter) barcoded liposomes were injected intravenously at a barcode concentration of 500 nM. After 48 h, the mouse was killed and its organs (+tumour) were sourced. In addition, a core biopsy was taken from live mice using an adjustable coaxial Temno biopsy device equipped with an 11-cm long, 20 G needle (San Diego, CA, USA). The average tissue biopsy volume was 40 mm^3^ (corresponding to approximately 40 million cells)^57^,^58^.

### Single-cell suspension

The tumour was held on ice-cold PBS until the beginning of tissue dissociation, always within less than 1 h from excising the tissue. Then, the tissue was dissociated enzymatically using 25 mg ml^−1^ hyaluronidase (Sigma-Aldrich, Rehovot, Israel), 25 mg ml^−1^ collagenase-3, 50 mg ml^−1^ collagenase-4 (Worthington Biochemical, Lakewood, NJ, USA) in 2 ml sample, 37 °C, and incubation of 40. The dissociation continues physically using a GentleMacs machine (Miltenyi Biotec, Teterow, Germany) based on mouse tumour dissociation protocol. Single-cell suspension was obtained by passing the suspension in 70 μm cell strainer (BD Biosciences, San Jose, CA, USA). The average number of cells that are sorted is about 5 million cells.

### Live and dead assay

After dissociating the tumour tissue into a single-cell suspension, the cells were counted and stained using PI. The PI intercalates with the DNA in intact dead cells that have sufficient permeable membrane. We found that intact dead cells with barcodes inside them are most effectively sourced from the tumour within 48 h after the administration of barcoded nanoparticles.

### DNA extraction from primary tumour or tissue

After sorting the cells, they were centrifuged (400*g*, 7 min). The supernatant was discarded and the modified Bligh and Dyer assay was performed (see above). The upper (aqueous phase) was concentrated using a Speedvac Concentrator (UNIVAPO 100H, UNIEQUIP, Fraunhofer, Germany) and the DNA barcodes were analysed using RT–PCR and sequencing.

### Bio-distribution of ICG-liposomes in subcutaneous 4T1 tumours

The experiment was carried out in 10-week-old female BALB/c mice (Harlan Laboratories Inc.). Murine breast cancer cell line, 4T1 (PerkinElmer, MA, USA), was injected subcutaneously into the rear right flank (5 × 10^5^ cells in 100 μl of PBS). Three weeks after the injection, 150 μl of 120 nm ICG liposomes or free ICG dye (0.09 mg ml^−1^) were injected intravenously through the mice's tail vein. The bio-distribution of particles or free dye was monitored under *In Vivo* Imaging System for a period of 72 h.

### *In vivo* therapeutic efficiency

Four groups (*n*=6 each) were divided as follows: control, cisplatin, doxorubicin and gemcitabine. Hundred microlitres of 10^6^ cells ml^−1^ of 4T1 (triple negative breast cancer) were injected subcutaneously to 10-week-old BALB/c female mice. The mice were weighed and the tumour dimensions were taken three times a week. The tumour volume was measured with a caliper (Digital-Messschieber mit Rolle und Datenausgang, MIB -Messzeuge GmbH, Germany and calculated as length/2 × (width)^2^. Therapeutic treatment began when the tumour volume reached ∼500 mm^3^, approximately 10 days after the initial tumour cells injection. All the therapeutic groups received a single dose each week as follows: doxorubicin 5 mg kg^−1^, cisplatin 6 mg kg^−1^ and gemcitabine 125 mg kg^−1^. The control group received 100 μl of saline.

### Fluorescence tissue microscopy

The slides of the paraffin-embedded tissue were scanned using a Pannoramic Midi Virtual Microscope (3D Histech, Hungary) equipped with a Turrets Chroma 49000/2/4-ET reflector and T-400/495/565 beam-splitter. The cell nuclei were stained with DAPI, DNA barcodes with fluorescein, and nanoparticles with rhodamine. The images were analysed using Pannoramic Viewer software (3D Histech).

### Histology

After killing the mice, the tumour was extracted and kept in 10% natural buffer formalin at room temperature. Later, the tissue was paraffin embedded. The slides from the tumours were stained with haematoxylin and eosin. In addition, the tissue slides were immunohistochemically stained with rabbit monoclonal anti-Ki67 antibody (SP6; Thermo Scientific, Fremont, CA, USA, diluted 1:300 in PBS) to determine the proliferation index.

The slides of the paraffin-embedded tissue were scanned and analysed using a Panoramic Midi virtual microscope (3D Histech) with a × 20 objective magnification, 30 ms DAPI exposure time, 700 ms EGFP and CY3 exposure time.

### Statistics

Differences between two means were tested using an unpaired, two-sided Student's *t*-test.

### Data availability

All data that support the findings of this study are available from the corresponding author upon request.

## Additional information

**How to cite this article:** Yaari, Z. *et al*. Theranostic barcoded nanoparticles for personalized cancer medicine. *Nat. Commun.* **7,** 13325 doi: 10.1038/ncomms13325 (2016).

**Publisher's note:** Springer Nature remains neutral with regard to jurisdictional claims in published maps and institutional affiliations.

## Supplementary Material

Supplementary Information Supplementary Figures 1-5 and Supplementary Methods

## Figures and Tables

**Figure 1:**
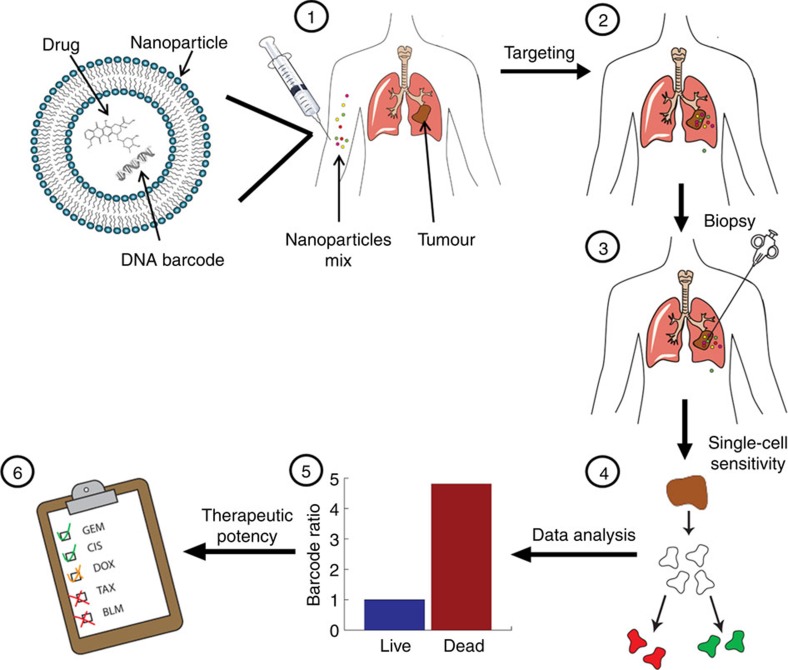
Using nanotechnology to probe the sensitivity of cancer to medicines. Barcoded nanoparticles (BNPs) were loaded with different drugs and corresponding DNA barcodes. (1) A cocktail of BNPs was injected intravenously. (2) The BNPs targeted the tumour and each of the drugs carried out its therapeutic activity inside different tumour cells. (3) Forty-eight hours later, a biopsy was taken from the tumour, and the biopsied tissue was dissociated into a single-cell suspension. (4) Each of the cells was sorted according to its viability (live/dead). (5) The barcodes were extracted from the live/dead cells and amplified using real-time PCR. The codes were detected by sequencing. The activity of the drugs inside the tumour was analysed by recording the number of each barcode found in the live cells, versus the number of barcodes found inside the dead cells. In this manner, the orthotropic tumour was used as a miniature laboratory, which was diagnosed with the nanoparticles at a cellular level. (6) Based on the screened results, a suggested treatment protocol was devised. In our studies, we found this predictive assay to achieve the best therapeutic results. The overall diagnosis takes less than 72 h.

**Table 1:**
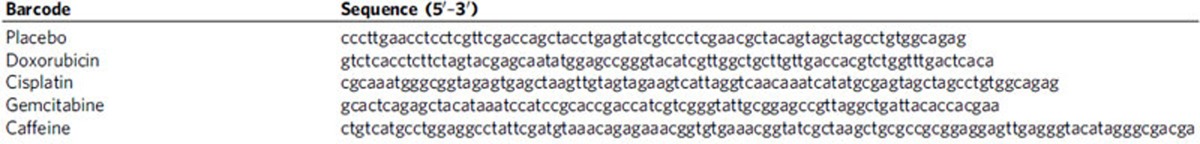
Barcode sequences.
