# Supplementary material for: Theranostic barcoded nanoparticles for personalized cancer medicine
Source: Nat Commun. 2016 Nov 10;7:13325. doi: 10.1038/ncomms13325 (PMC5109543; doi:10.1038/ncomms13325)
Supplement: Supplementary Information — Supplementary Figures 1-5 and Supplementary Methods [file ncomms13325-s1.pdf]

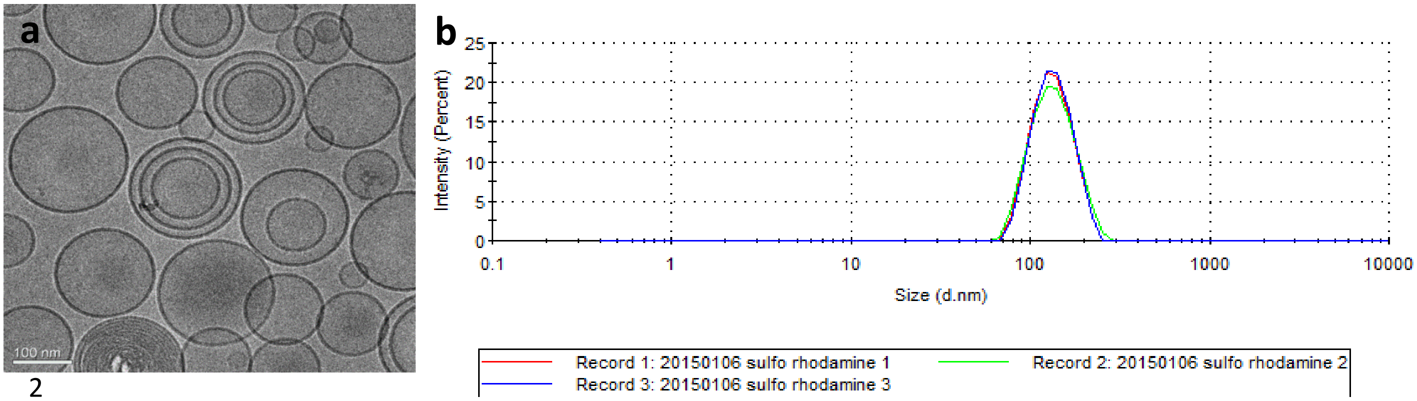

Supplementary Figure 1. CryoTEM images of the barcoded nanoparticles (a) and size measurements of the particles (b). Liposomes were loaded with DNA barcodes and were imaged using cryo-TEM and measured for their size using dynamic light scattering (Malvern Zetasizer ZSP), mean size was 120 nm and the polydispersity index (PDI) was ~0.07.

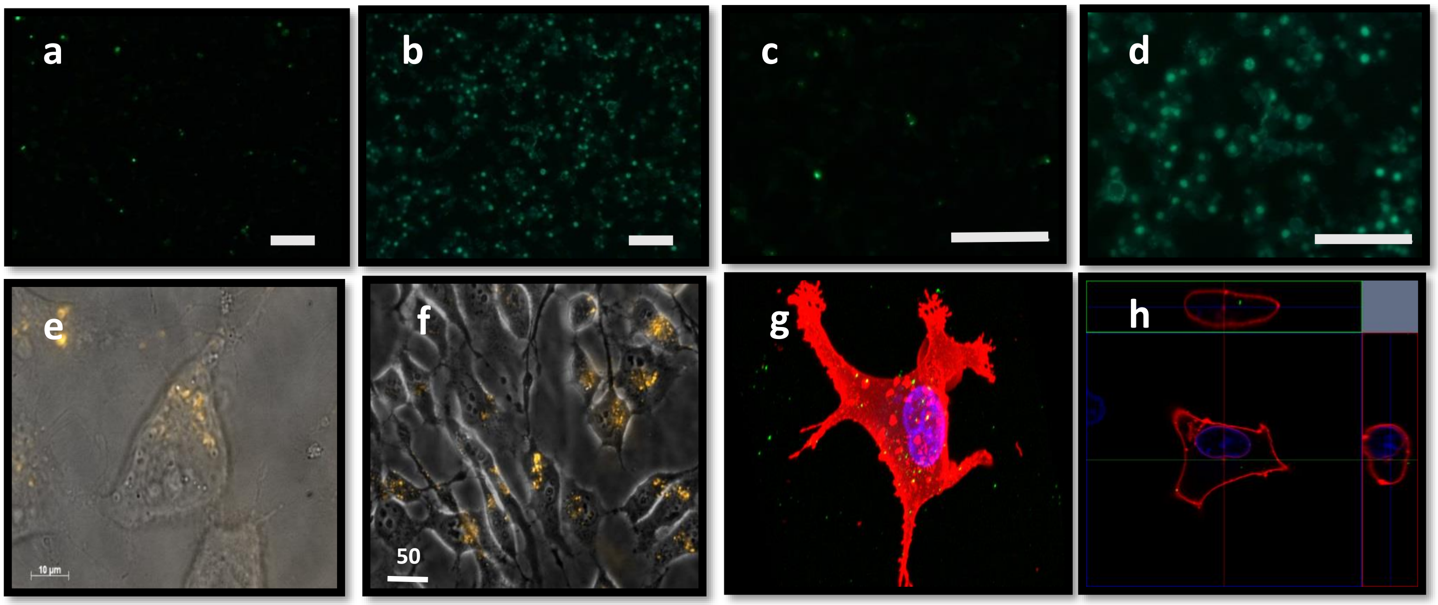

Supplementary Figure 2. The uptake of encapsulated and non-encapsulated barcodes by cancer cells.

Nanoparticles carry compounds that do not cross cellular membranes into cells. To distinguish between drugs that entered the cell and those outside the cell, we chose DNA barcodes that are impermeable to the cell without the carrier. To test the uptake of the free and encapsulated barcode, 4T1 breast cancer cells were incubated with each of these components, and then imaged using confocal microscopy. (a-d) Fluorescent microscopy images of 4T1 cells that were incubated with free (non-liposomal) fluorescently-labeled DNA barcodes X10 (a), X20 (c), or with liposomes containing fluorescently labeled DNA barcodes. X10 (b), x20 (d) magnifications. Scale bar is 100µm.

Barcoded nanoparticles were taken up by cancer cells. (e,f) Fluorescent microscope images of MDA-MB-231 cells incubated with rhodamine-labeled liposomes X100 (e), X40 (f) enlargements. (g,h). Confocal microscopy images of barcoded nanoparticles inside 4T1 breast cancer cell. The cellular membrane is stained red (DID), the nucleus blue (HOECHST), and the DNA barcodes green (FITC). 3D model of the cell (g), side sections of the cell (h).

Supplementary Figure 2a-d, shows that DNA does not penetrate cells without a carrier, in comparison to the vast uptake of the liposome encapsulated DNA. This is due to the negative charge on the DNA molecule that cannot penetrate the lipid membrane. In contrast, the liposome ferries the encapsulated DNA across the membrane.

In addition, the uptake of the liposomes by the cells was studied using fluorescently labeled lipids (16:0 Liss Rhodamine PE). After seeding cells, incubating them with the liposomes and washing, the cells were imaged (Cell Observer, Zeiss). Supplementary Figure 2e,f shows the uptake of barcoded liposomes by breast cancer cells.

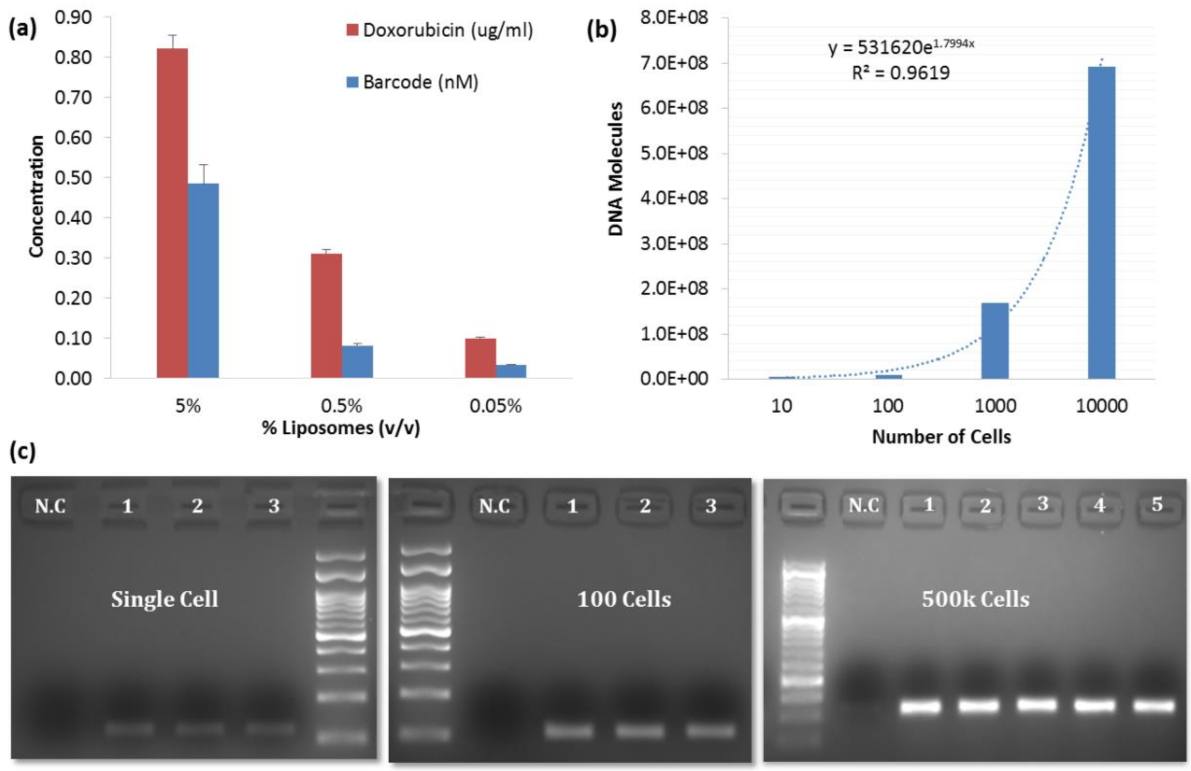

Supplementary Figure 3. The uptake of barcoded nanoparticles by cancer cells. A direct correlation between the barcode and doxorubicin concentrations after extraction both from 500k cells (a), a correlation between barcode copies and number of cells (b). The cells were counted by FACS (b), or diluted several times (c).

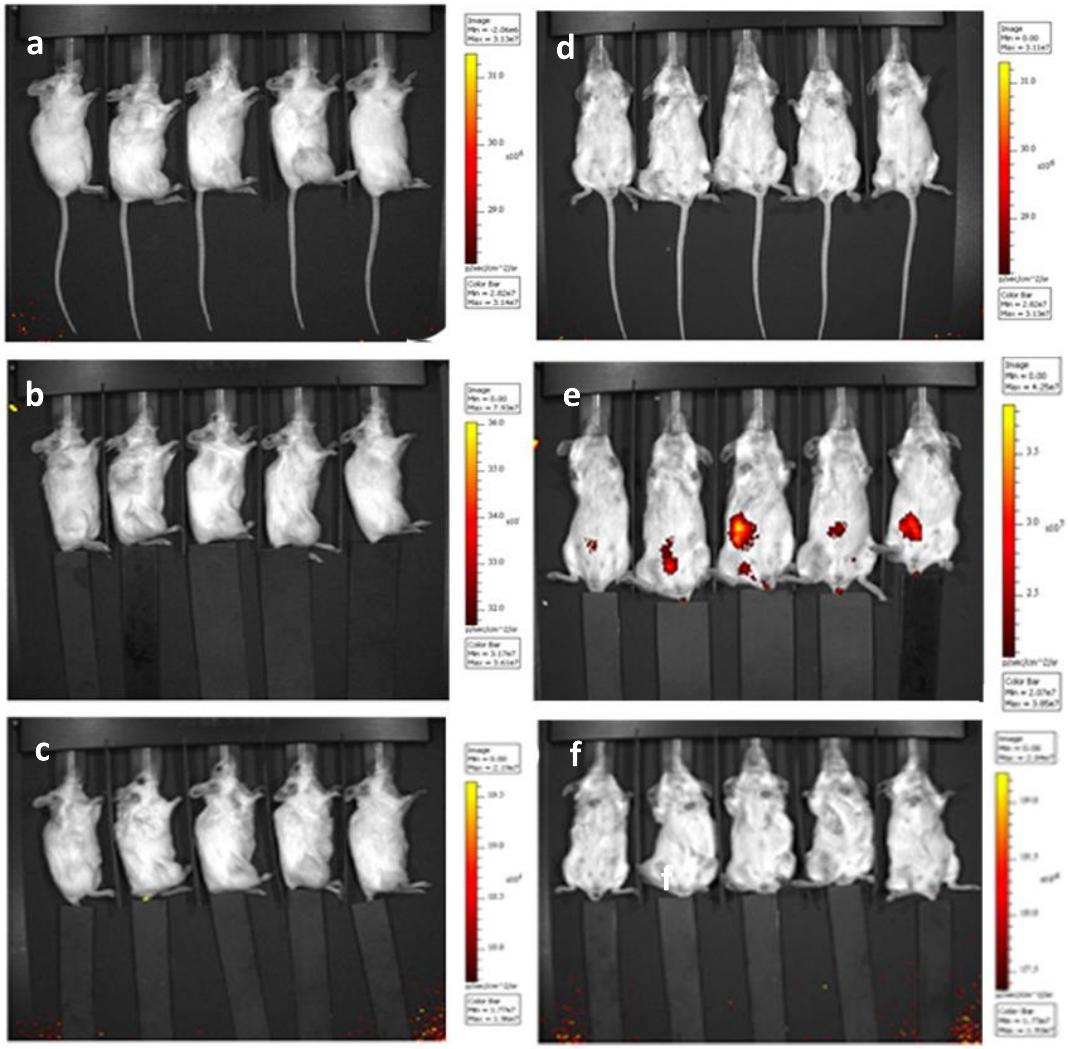

Supplementary Figure 4: Biodistribution of <u>free</u> ICG dye in mice bearing triple-negative breast cancer tumors. Free ICG (0.09 mg/ml) was injected IV to tumor bearing mice and the mice were imaged using an IVIS whole animal fluorescent imaging system; before injection (a,d); after 24 hours (b,e) and 48 hours after the injection (c,f). (a-c) images are in lateral position while (d-e) images are in axial position. The left mouse is the control.

While at the lateral position no dye was detected at all the time periods (Supplementary Fig. 4a-c), in the axial position the dye was observed mainly in the liver and the bladder tissues (Supplementary Fig. 4e). 48 hours after the injection, no free dye could be detected in the mice.

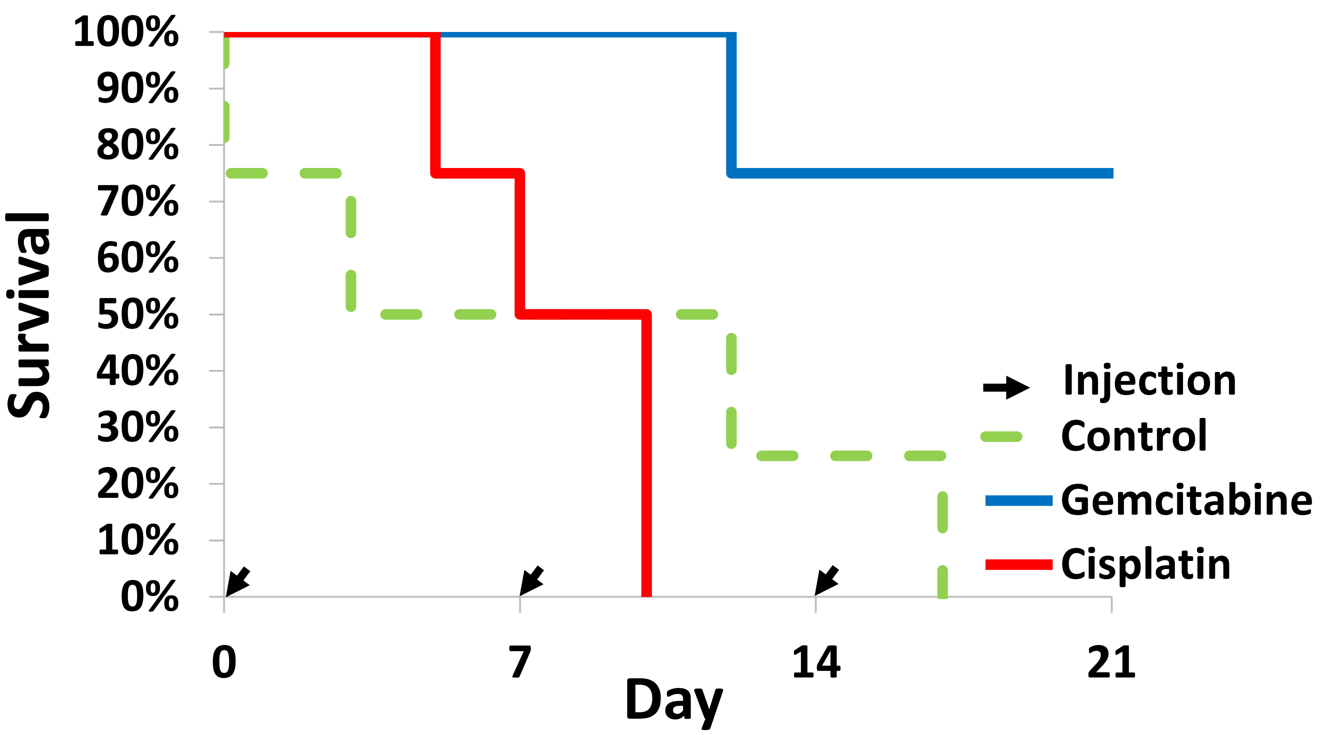

Supplementary Figure 5. Kaplan-Meier analysis of animal survival following the therapeutic treatment. The in vivo efficacy of the treatment mirrored the diagnostic prediction, the life span was prolonged in the group that received Gemcitabine. Animal end-point survival was defined as tumor diameter greater than 1.5 cm.

<u>Supplementary Methods</u>

CryoTEM. CryoTEM was performed as follows: lipid dispersions at concentration of 5 mM are prepared in a controlled-environment vitrification system at 25 °C and 100% relative humidity and examined in a Philips CM120 cryo-electron microscope operated at 120 kV. Specimens were equilibrated in the microscope below –178 °C, then examined in the low-dose imaging mode to minimize electron beam radiation damage, and recorded at a nominal under-focus of 4–7 nm to enhance phase contrast an Oxford CT-3500 cooling holder was used. Images were recorded digitally by a Gatan MultiScan 791 CCD camera using the Digital Micrograph 3.1 software package (Gatan).
